# Religious Experience as a Predictor of the Meaning in Life and Life Satisfaction in the Lives of Polish Women after a Stillbirth

**DOI:** 10.1007/s10943-022-01698-z

**Published:** 2022-11-23

**Authors:** Stanisław Głaz

**Affiliations:** Jesuit University Ignatianum in Krakow, Ul. Kopernika 26, 31-501 Kraków, Poland

**Keywords:** Religious experience, Satisfaction with life, Meaning in life, Stillbirth, Polish women

## Abstract

Many researchers have demonstrated the relationship of religiosity with dimensions of mental health such as searching for meaning in life, the presence of meaning in life, and life satisfaction. Hence, this study attempts to link such aspects of religiosity with the experience of the presence and/or absence of God with the search for and presence of meaning in life and satisfaction with life among a group of Polish women who have experienced a stillbirth in the past five years. The study included 64 women who lost a baby through stillbirth after the 22nd week of gestation. All the women were born into Christian families and declared themselves to be Christians and actively practicing their faith. Women’s ages ranged from 29 to 47 years. The research results showed that the experience of God’s presence and absence have a positive and significant relationship with the search for meaning in life and the presence of meaning in life, as well as with the satisfaction with life in the lives of women after stillbirth. The strongest relationship was between the presence of meaning in life and life satisfaction (*r* = .72; *p* < .01), God’s presence and life satisfaction (*r* = .66; *p* < .01), as well as the presence of meaning in life and the search for meaning in life (*r* = .57; *p* < .01). The structural equation model showed that the experience of God’s presence and God’s absence have a significant, *direct* impact on the search for meaning in life and the presence of meaning in life, and the satisfaction with life. These also have an *indirect* impact on the satisfaction with life in the lives of women who have lost a child to stillbirth.

## Introduction

Stillbirth is defined as the death of a baby during a woman’s pregnancy. The accepted criteria for stillbirth vary in many countries (Lawn et al., 2010; van den Akker, 2011). World Health Organization (WHO) determined that a baby who dies at 28 weeks or later is classified as stillbirth. Under Polish law, pregnancy loss after the 22nd week is considered a stillbirth. (Dziennik Ustaw, 2020 pt. 666).

Death and birth are the two most significant events in a Woman’s life (Burden et al., 2016). Women who have experienced stillbirth exhibit post-traumatic symptoms. Stillbirth has a devastating impact not only on the mother but also on family members and their social engagement (Gold et al., 2017; Gravensteen et al., 2018). It involves extremely difficult spiritual and personal experiences that sometimes have a lasting impact on the religious life of the mother and family (Gravensteen, 2017; Lamb, 2002).

Children whose siblings died during the mother’s pregnancy perceived their parents as more controlling of the family situation, and overprotective, which limited their initiative and activity than parents who did not lose a child (Pantke & Slade, 2006). The children perceived the behavior of their parents after the loss of the child as inadequate to the situation. As a result, symptoms such as the lack of security sense in the family, loss of trust in others, and lack of openness to peer environment appeared in their lives (Pilecka, 2004). On the other hand, in recalling events and experiences preceding, accompanying, and following the diagnosis and stillbirth, the bereaved parents manifested specific attitudinal characteristics. They were concerned with the maintenance of hope, the importance of the child’s personality, care for the child, and relationships, some positive but most negative (Nuzum et al., 2018).

The loss of a child was often associated with giving up one’s previous lifestyle and future (Pilecka, 2004). DeFrain’s (1991) research indicated that over 30% of women experiencing stillbirth had suicidal thoughts. Some women tried to commit suicide. The death of a child was often a source of spiritual distress as well as a challenge for parents (Cowchock et al., 2010). The experience of stillbirth encompasses many areas of a woman’s life and results in a great deal of loss, loss of self-esteem, loss of parental aspirations, and the emergence of anxiety about the possibility of creating another life (Lamb, 2002). Findings have shown that stillbirth is a depressing experience in women’s lives, causing high levels of psychological symptoms including depression, anxiety, fear, negative well-being, and psychosocial consequences (Cacciatore & Ong, 2012; Vance et al., 1991). Postpartum women who placed a high value on pregnancy experienced the loss of their child more strongly than women who placed less value on pregnancy (Sikora, 2014).

Research conducted by Guzewicz and the team, based on Gough and Heilbrun’s List of Adjectives, found that Polish women who had lost a child chose fewer positive adjectives to describe themselves in their current situation than women who had not such an experience. They feared for the future and were overwhelmed by the events of their lives. They had a lower need to understand themselves and others and to care about others. These women are anxious, tense, and overwhelmed by their situation, so they do not function effectively, have low self-confidence, and give in to others. They tend to demean themselves and lose self-confidence. They are less interested in interpersonal interactions; are less friendly, gentle, tolerant, and sensitive. They have lower levels of energy, entrepreneurship, and determination, and are less creatively active. They have less need to interact with the opposite sex and do not feel the need to share their experiences (Guzewicz et al., 2014). Another study by Guzewicz (2014) suggests that the most common symptoms in women’s lives after the loss of a child were feelings of guilt, lowered mood, pessimism, tearfulness (affective domain), irritability in relationships with others (social domain), inability to work, sleep disturbances, and fatigue (somatic domain).

In a group of Taiwanese mothers who experienced stillbirth (Hsu et al., 2002), four main methods regarding coping processes were identified. Most often, women are aimed at transforming the meaning of death, doing something for the lost child, expressing readiness for another pregnancy, and rebuilding a social relationship. Polish women after the loss of a child most often experienced support from their husbands while engaging in work and focusing on the future (Sikora, 2014). Burden’s (2016) research found that parents who experienced the tragedy of stillbirth were simultaneously able to strengthen mental resilience and develop new life skills and abilities.

Results among Australian couples bereaved after stillbirth demonstrate that negative emotions had an intrapersonal relationship with bereavement in men and both an intrapersonal and interpersonal relationship with bereavement in women and that the average level of guilt correlated with the low level of bereavement and low level of sadness in women (Barr, 2012). Women who experienced a stillbirth showed more intense grief reactions than women who miscarried (Cuisinier et al., 1993), and grief symptoms were exacerbated by a lack of support from society, pre-existing relationship difficulties, and absence of surviving children (Kersting & Wagner, 2012; Pilecka, 2004). A study of bereaved mothers in Michigan who lost a child found that mothers were most likely to feel guilt or self-blame after the loss. Post-loss women had higher levels of grief than men 6–8 weeks after losing a child. However, this difference decreased at 1 and 2 years after child loss (McIntosh et al., 1993). On the other hand, religious and practicing women are better able to cope with grieving the loss of a child than women who are not religiously committed (Kersting et al., 2007).

With the increase in depression and interpersonal violence, the sense of guilt in women’s lives has increased (Gold et al., 2017). 26% of the women surveyed admitted that they were the ones most to blame for losing their child. Self-blame, in particular, has been positively correlated with symptoms of anxiety and depression (Cacciatore et al., 2013). It has also been found that self-blame often prompts women to repair the loss they have suffered (Weinberg, 1995). Lower self-esteem of mothers after child loss led to a higher level of stress (Bödecs et al., 2011). Whereas, research among African-American and Latina women shows that women who experienced stillbirth or child death had lower self-esteem than women who did not lose a child (Wonch et al., 2017). Some studies report that women’s life satisfaction increases with the timing of pregnancy (Dyrdal et al., 2011), but then declines in the first years after birth to pre-pregnancy levels, and also declines in those who miscarry (Gravensteen et al., 2018). Stillbirth reduces mothers’ sense of life satisfaction and appreciation from others, and single women were more likely to experience social failure after stillbirth than those who experienced the loss of a child in a marital relationship (Giannandrea et al., 2013; Rådestad et al., 1997). Women who have experienced a miscarriage are less satisfied with welfare than women who have had a stillbirth (Cuisinier et al., 1993).

The fact of stillbirth has been interpreted by women believers in many ways. Ultra-Orthodox Israeli women sought strength in religious faith. Fetal loss was experienced as a test of their faith in God and seen as a way to experience God’s love. In such a situation, most often the women’s faith was strengthened and brought relief, as well as peace and trust in God as the giver of life. In addition, the findings indicate that the respondents did not direct their anger over the loss of their child to God, and sometimes perceived the negative feelings as a threat to their faith (Hamama-Raz et al., 2014). Iranian women believe that their dead children have a great chance to live in another world and they will be returned to their parents in a changing world (Nematti, 2005). Polish women who had lost a child presented a negative attitude toward God and a low level of relationship with Him and blamed Him for the loss of the child. Some parents who have lost a child have often been convinced that God caused their loss (Downey et al., 1990; Guzewicz et al., 2014). Other women believe that their dead children are in a safe place (Kalu, 2019). The authors of the study suggest that this is probably the result of blaming God for the loss and seeing it as God’s punishment for sins.

After losing a child, women often expect social, moral, and spiritual support. Many women reported that their spiritual needs were not adequately met during their hospital stay (Nuzum et al., 2017b). Most often, they received assistance from the chaplain. His presence in the hospital, attentive listening, and the liturgy he provided helped them understand a painful event (Newitt, 2014). They most often expected pastoral tenderness and empathetic presence from the chaplain and viewed these as key attributes of spiritual care (Nuzum et al., 2017a). Women could also rely on their husbands, partners, and relatives for support (Bubiak et al., 2014; Miernik, 2017). On the other hand, Olson’s (2013) research shows that health professionals do not always adequately meet the needs of women and their families during hospitalization after the loss of a child. Many health professionals feel that they have not been adequately trained to provide optimal care to parents experiencing child loss (Emond et al., 2019).

Women following the loss of a child who attended organized religious activities at least several times a month were significantly less likely to have high rates of depressive symptoms (Mann et al., 2008), and women who had experienced a stillbirth and who regularly attended church, seeking spiritual comfort in prayer, had lower levels of depression and anxiety than women who attended irregularly. In addition, the frequent participation of parents who had experienced the loss of a child in church liturgy was indirectly associated with their improved well-being and lower anxiety (Thearle et al., 1995).

The association of two components of religiosity: religious participation and religious meaning, with three coping process variables (perceived social support, cognitive processing of loss, and finding meaning in death) in the lives of women who have lost a child was shown. Greater religious participation was associated with greater social support perception and with greater importance of finding meaning in loss, and the importance of religion was positively related to cognitive processing and finding meaning in death (Thearle et al., 1995).

## Research Problem and Hypotheses

Stillbirth can be diagnosed by ultrasound examination to show that the baby’s heart is no longer beating. Research indicates that the most common causes of stillbirths are infections, birth defects, and pregnancy complications, such as pre-eclampsia (Bubiak et al., 2014; Chazan, 2013).

In Poland, between 2017 and 2019, according to statistics (Central Statistical Office), each year about 1,700 women gave birth to a stillborn child, which is about 0, 3% of all pregnancies. Losing a child to stillbirth is undoubtedly a difficult event for the parents, sometimes for the entire family. Painful experiences are compounded when the child was particularly desired by the parents. In this situation, stillbirth becomes a family event affecting each family member in their own way (Campbell-Jackson & Horsch, 2017; Guzewicz et al., 2014).

Events such as death, illness, and stillbirth and their consequences are most often perceived as traumatic, less often as a life challenge (Roberts & Montgomery, 2015). They can be an opportunity to achieve not only internal freedom but also internal maturity. Such events are often treated as a sign of maturity (Frankl, 2000; Popielski, 2008). They can support the strengthening of mental health and religiosity, which is manifested in the search for meaning in life, shaping a more mature identity, undertaking social activities for other people, and caring for the development of one’s own religiosity (Krok, 2017; Okła, 2009). On the other hand, giving too much attention to the negative effects of traumatic events often leads to deterioration and weakening of mental health and religiosity, which manifests itself in a sense of threat to one’s own identity, low self-esteem, guilt, weakening of interpersonal relations, spiritual and moral struggles (Guzikowski, 2009; Nuzum et al., 2016; Podolska, 2011), and sometimes even in suicide attempts (Pilecka, 2004).

According to many researchers, mature religiosity in the life of believers has a meaning-creating character (Koenig, 2012; Merrill et al., 2009; Pargament, 1997; Prusak et al., 2021). However, it can also be a source of tension and spiritual struggle sometimes (Exline & Rose, 2005; Roesch & Ano, 2003). Research shows that religiosity has a positive impact on the mental health of people affected by traumatic experiences, such as cancer (Charzyńska, 2017; Krok et al., 2019), death of a loved one (Buksik, 2003), suffering (Frankl, 2000), stillbirth (Hamama-Raz et al., 2014; Mann et al., 2008). People who experienced these kinds of events in their lives often prayed and made use of various religious practices. Faith and religious practice were important sources of support for them. Religiosity helped them find comfort and understand the meaning of painful experiences (Krok, 2015; Mann et al., 2010).

There is a visible lack of research among women who had a stillbirth. Losing a baby in the prenatal period is a reversal of the natural process of life and poses a threat to a woman’s sense of meaning in life and life satisfaction. Religious coping strategies can be an effective help. Therefore, it seems justified to show the relationship between the two dimensions of human life related to the mental health and the religiosity of women who have had a stillbirth, such as a) the experience of God’s presence, which suggests that a person experiencing God’s presence has trust in God and is open to the needs of others, perceives her life as meaningful and valuable, and the experience of God’s absence, which suggests that a person experiencing God’s absence tends to trust God more, to get to know oneself better and to be more open to others (Głaz, 2021), b) sense of the meaning in life, which concerns the way of understanding and positively interpreting the meaningfulness of life events, as well as exceeding the existing reality (Frankl, 2000; Popielski, 2008). The sense of the meaning in life concerns the presence of the meaning in life, which means that a person understands what is conducive to her, to make her life more creative, it also concerns the search for the meaning in life, i.e., it includes an appropriate effort to discover what would make human life more meaningful (Steger et al., 2006); c) satisfaction with life, which covers the cognitive sphere of the individual, and concerns their aspirations, own tasks, a realization of own aspirations, and relates to the emotional sphere, which significantly affects the feeling of positive emotional experiences or the lack of them (Diener & Chan, 2011; Diener et al., 1985).

Shows the relationship between the religious experience of God’s presence and God’s absence, the presence of the meaning in life and the search for the meaning in life, and the life satisfaction of women after stillbirth, who grew up in a Christian family and consider themselves believers and practitioners, can be an important supplement to previous analyzes. It has been suggested that in the lives of women after the loss of a child, God’s presence and God’s absence play a positive role and serve as a predictor of the search for meaning in life, the presence of meaning in life, and life satisfaction. Moreover, it is assumed that women, after losing a child, show a great tendency to search for the meaning in life and a specific interpretation of the event that has occurred and that the sense of the meaning in life and the search for the meaning in life play a mediating and positive role between the religious experience of God’s presence and God’s absence, and life satisfaction.

Based on the literature and previous research, the following research hypotheses were developed.

### Hypothesis 1

Women after stillbirth have higher levels of God’s presence than God’s absence, higher levels of seeking meaning in life than the presence of meaning in life, and satisfaction with life.

### Hypothesis 2

There is a stronger and more positive association of God’s presence than God’s absence with life satisfaction in women’s lives after stillbirth.

### Hypothesis 3

In a woman’s life after stillbirth, there is a stronger and more positive relationship between God’s presence and God’s absence with the search for meaning in life than with the presence of meaning in life.

### Hypothesis 4

The search for the meaning in life and the presence of the meaning in life plays the role of a mediator between God’s presence and God’s absence, and life satisfaction in the lives of the woman after stillbirth.

## Methods

### Sample and Procedure

Outreach to women after stillbirth was made to those working in psychological and pastoral care centers in northern Poland. The study received ethical approval from Jesuit University Ignatianum. An information leaflet was then provided which indicated the purpose of the study and encouraged women to complete the questionnaires accurately. The recruitment process involved the women agreeing to participate in the study, which was completely anonymous. Eighty-seven women were approached to participate in the study, and sixty-four agreed to participate, so the response rate was almost 74%. All the participants provided written informed consent to complete the questionnaire. Each woman was given contact information if one of them needed additional support. Several women reported that they needed it and took advantage of the psychological and pastoral support offered.

Finally, the sample consisted of sixty-four women who had experienced the loss of a child through a stillbirth that occurred after the 22nd week of pregnancy (Table 1). The group of women participating in the study was those who had lost a child within 5 years prior to testing. The age of the women ranged from 29 to 47 years (*M* = 41.87; *SD* = 6.31).

**Table 1:** Basic characteristics of women

Variables	Category	*N*	Percentage (%)
Sex (female)		64.0	100
Current place of living	Village	24.0	37.5
	City	40.0	62.5
*Civil status*	Married/in marital-like relationship	35.0	54.7
	Divorced/currently single	11.0	17.2
	Separated	9.0	14.1
	Single	7.0	10.9
	Widowed	2.0	3.1
Religion Education	Catholic	64.0	100
	University education	47.0	73.5
	Secondary education	13.0	20.3
	Primary school	4.0	6.2
Offspring	Without children	9.0	14.1
	One child	22.0	34.3
	Two or more	33.0	51.6
Stillbirth	Once	27.0	42.2
	Twice	20.0	31.2
	Three times	17.0	26.6
Support	Family	21.0	32.8
	Friends	20.0	31.2
	Psychologist	11.0	17.2
	Priest	7.0	11.0
	Support group	5.0	7.8

### Tools

Survey participants completed three instruments that measure religious experience, sense of meaning in life, and satisfaction with life.

The Intensity of Religious Experience Scale (IRES). It was developed by Głaz (2021). It is used to examine religious experience, that is, the experience of the presence of God and the absence of God of a Christian nature. It consists of 18 statements. The first factor, which includes 10 statements, concerns the experience of God’s presence (GP). It contains items like: “The experience of God’s presence is conducive to deepening my religious faith”, “The experience of God’s presence is conducive to deepening the intimacy with Him”, and "Thanks to the experience of the presence of God, the lives seems to be more meaningful and valuable", “During the inner experience of God’s presence, I experience states of deep peace and joy”. The second factor, which consists of 8 statements, relates to the experience of God’s absence (GA). It contains items like: “Experiencing God’s absence is conducive to deepening my trust in Him”, “During the experience of God’s absence, I have the opportunity to better understand myself", and "Experiencing God’s absence enriches my religious life". Each assertion contains seven possible answers. The response is placed on a Likert-type scale (1 to 7) indicating how much the respondent agrees or disagrees with the statement. 7. *strongly yes* (strongly agree), 6. yes (agree), 5. *rather yes* (rather agree), 4. *can’t decide*, 3. *rather no* (rather disagree), 2. *no* (disagree), 1. *strongly no* (strongly disagree). The extracted factors are correlated among themselves. The obtained correlation coefficient between the extracted factors is at a medium and positive level. The reliability of the scale was estimated using the internal consistency method, where Cronbach’s *α* coefficient for the subscale of experiencing the presence of God was 0.93, and for the subscale of experiencing the absence of God was 0.86.

The Meaning in Life Questionnaire (MLQ). The MLQ questionnaire measures the meaning in life from two-time perspectives, present, and future (Steger et al., 2006). The two-time perspectives are complementary, as researchers assume that both the presence of meaning in life and the search for meaning are important for assessing the meaning of an individual’s entire life. The questionnaire consists of 10 questions to which a 7-point Likert scale is assigned (from absolute untruth = 1) to absolute truth = 7). Questions in the MLQ-P (Presence) subscale refer to the presence of meaning in life–they measure the declared, obtained meaning in life and its realization. On the other hand, questions in the MLQ-S (Search) subscale refer to the search for meaning in life, to the need for continuous meaning and purpose in the life of a person. The tool was adapted to Polish conditions by Kossakowska, Kwiatek, and Stefaniak (2013). Cronbach’s *α* coefficient as an indicator of the reliability of the questionnaire for the ten test items was 0.79. For the subscale presence of meaning in life, Cronbach’s α coefficient was 0.86, and for the subscale searching for meaning in life 0.72.

The Satisfaction with Life Scale (SWLS) by Diener, Emmons, Larsen, and Griffin (Diener et al., 1985). It is a commonly used instrument that measures life satisfaction as a conscious and cognitive evaluation of one’s own life, during which the individual compares the conditions of their life with self-imposed standards. The scale contains 5 statements to which the person surveyed gives an answer on a 7-point scale. If the result of the comparison, indicating the general degree of satisfaction with one’s own life, is satisfactory, this results in a feeling of satisfaction with one’s own life. The higher the score obtained, the higher the feeling of satisfaction with the life of the person. The scale can be used individually or in groups. The reliability of the scale measured by the test–retest method was 0.83, while Cronbach’s *α* reliability coefficient was 0.72. The scale was adapted to Polish conditions by Juczyński (2001). The tool in the Polish version has satisfactory psychometric indices. The Cronbach’s *α* reliability coefficient is 0.81, and the theoretical accuracy of the tool was checked by factor analysis, which confirmed the existence of one factor.

### Statistical Analysis

Descriptive statistics were presented (*M* and *SD*) for the analyzed variables. Multiple regression analysis was used. The *R*^*2*^ coefficient was used to determine the percentage of the variance of the dependent variable explained by the independent variables. Pearson’s *r* correlation coefficient was calculated to determine the strength of the relationship and its nature between the variables included in this study. Moreover, structural equation techniques were used to show more complex relationships between variables.

## Results

In order to verify the research hypotheses, the analysis of data obtained in the Intensity of Religious Experience Scale (IRES), the Meaning in Life Questionnaire (MLQ), and the Satisfaction with Life Scale (SWLS) among religious and practicing women who grew up in a Christian family and experienced the loss of a child were used.

The result obtained in the Intensity of Religious Experience Scale (IRES) (Table 2) shows that women after stillbirth exhibit a high level of God’s presence (*M* = 5.4; *SD* = 0.968) and an average level of God’s absence (*M* = 4.6; *SD* = 1.023).

**Table 2:** Descriptive statistics for variables obtained in the Intensity of Religious Experience Scale (IRES), the Meaning in Life Questionnaire (MLQ), and the Satisfaction with Life Scale (SWLS) for women after stillbirth

Variables	*M*	*SD*	Cronbach’s *α*
PG	5.4	.968	.880
AG	4.6	1.023	.872
MLQ-P	5.2	.928	.779
MLQ-S	4.6	.972	.599
SWLS	4.7	1.102	.867

*PG* = God’s presence, *AG* = God’s absence, *MLQ-P* = Presence of meaning in life, *MLQ-S* = Search for meaning in life, *SWSL* = Satisfaction with life

As one can see in Table 2 scores achieved in the Meaning in Life Questionnaire (MLQ) prove that women present a high level of presence of meaning in life (*M* = 5.2; *SD* = 0.928) and an average level of search for meaning in life (*M* = 4.6; *SD* = 0.972), but the result extracted in the Satisfaction with Life Scale (SWLS) shows that women have an average level of satisfaction with life (*M* = 4.7; *SD* = 1.102).

In the life of women after stillbirth (Table 3), God’s presence (PG) positively correlates with God’s absence (AG) (*r* = 0.46), presence of meaning in life (MLQ-P) (*r* = 0.48), search for meaning in life (MLQ-S) (*r* = 0.55), and with satisfaction with life (SWSL) (*r* = 0.66). Similarly, God’s absence (AG) positively correlates with the presence of meaning in life (MLQ-P) (*r* = 0.31), search for meaning in life (MLQ-S) (*r* = 0.33), and with satisfaction with life (SWSL) (*r* = 0.43). However, the presence of meaning in life (MLQ-P) positively correlates with search for meaning in life (MLQ-S) (*r* = 0.57) and with life satisfaction (SWLS) (*r* = 0.72), but search for meaning in life (MLQ-S) positively correlates with life satisfaction (SWLS) (*r* = 0.54).

**Table 3:** *r*-Pearson correlation coefficients received between variables obtained in the Intensity of Religious Experience Scale (IRES), the Meaning in Life Questionnaire (MLQ), and Satisfaction with Life Scale (SWLS) for women after stillbirth

Variables	AG	MLQ-P	MLQ-S	SWLS
PG	.46**	.48**	.55**	.66**
AG	–	.31*	.33*	.43**
MLQ-P	–	–	.57**	.72**
MLQ-S	–	–	–	.54**

*PG* = God’s presence, *AG* = God’s absence, *MLQ-P* = Presence of meaning in life, *MLQ-S* = Search for meaning in life, *SWSL* = Satisfaction with life

^**^p < .01; *p < .05

In the group of women after stillbirth (Table 4), God’s presence (PG) (*β* = 0.29), and God’s absence (AG) (*β* = 0.31), have a significant and positive relationship with presence of meaning in life (MLQ-P) (*R* = 0.52; *F* = 3.93; *p* = 0.0002. They explain 28% of the variance of this variable. Similarly, God’s presence (PG) (*β* = 0.38), and God’s absence (AG) (*β* = 0.37), have a significant and positive relationship with the search for meaning in life (MLQ-S) (*R* = 0.61; *F* = 3.11; *p* = 0.01). They explain 39% of the variance of this variable.

**Table 4:** Regression analysis results.

Variables	*b*	*β*	*t*	*p*	*R*^2^	
Dependent	Independent					
MLQ-P	PG	.41	.29	2.65	.01	.28
	AG	.29	.31	3.84	.009	
MLQ-S	PG	.42	.39	2.05	.04	.39
	AG	.49	.37	3.33	.002	
SWSL	PG	.51	.41	3.89	.008	.69
	AG	.29	.39	4.99	.001	

Influence of God’s presence (*PG*) and God’s absence (*AG*) on the presence of meaning in life (*MLQ-P*) and searching for meaning in life (*MLQ-S*), and on satisfaction with life (*SWSL*) for women after stillbirth

In addition, in the life of women after stillbirth (Table 4), God’s presence (PG) (*β* = 0.41), and God’s absence (AG) (*β* = 0.39), have a significant and positive relationship with life satisfaction (SWLS) (*R* = 0.82; *F* = 7.21; *p* = 0.001). They explain 69% of the variance of this variable.

Correlation and regression analysis performed on variables among women after stillbirth provided many interesting observations. To show the share of indirect variables in a given model, the method of structural equations was used. The presence of meaning in life and the search for meaning in life played the role of a mediator between the religious experience of God’s presence and God’s absence, and life satisfaction. The following indicators were used to assess the accuracy of fitting the data to the hypothetical model: CMIN/*df*, GFI, CFI, AGFI, and RAMSEA. Referring to the theoretical assumptions (Krok, 2017; Popielski, 2008) and the current results, an appropriate model was built. The model covers all the analyzed variables obtained among women who lost a child. The independent variables were the presence of God (GP) and the absence of God (GA), while the mediating variables were the presence of the meaning in life (MLQ-P) and the search for the meaning in life (MLQ-S), and the dependent variable life satisfaction (SWLS). It was found that the model fit rates were within the acceptable limits (CMIN/*df* = 1.81; GFI = 0.95; AGFI = 0.92; CFI = 0.94; RMSEA = 0.062) (Bentler & Bonett, 1980). The indicated model was adopted as explaining the relationships between the analyzed variables.

**Fig. 1:**
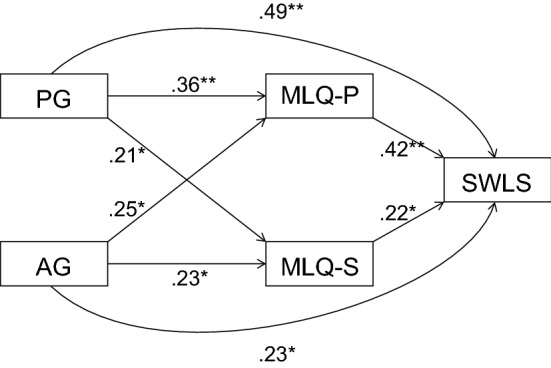
The relationship’s path diagram of God’s presence (PG), God’s absence (AG) and search for meaning in life (MLQ-S), presence of meaning in life (MLQ-P) with satisfaction with life (SWLS). **p < .01; *p < .05; numbers show the *β’s* regression coefficients

The results of the obtained dependencies are shown in figure (Fig. 1). The analysis shows that the experience of God’s presence (*β* = 0.49; *p* = 0.01) and God’s absence (*β* = 0.23; *p* = 0.05) have a significant and direct impact on the satisfaction with life, and indirect. The presence of God (*β* = 0.36; *p* = 0.01) and the absence of God (*β* = 0.21; *p* = 0.05) have a significant and direct influence on the presence of meaning in life and the search for meaning in life, which are mediation variables. Moreover, both mediators, the presence of meaning in life (*β* = 0.42; *p* = 0.01) and the search for meaning in life (*β* = 0.22; *p* = 0.05) have a direct and significant impact on life satisfaction.

## Discussion

The purpose of the article was to show the relationship between the religious experience of God’s presence and God’s absence, the searching for meaning in life and the presence of meaning of life, and life satisfaction in a group of believing and practicing Polish women who experienced a stillbirth. The hypotheses were verified and several important conclusions were identified.

The first hypothesis, which suggests that after stillbirth women have a higher level of God’s presence than God’s absence, and a higher level of searching for meaning in life than the presence of meaning in life and life satisfaction was only partially confirmed. The first part of the hypothesis has been confirmed. Women who have experienced stillbirth have a higher level of God’s presence than God’s absence. In this regard, the current research has confirmed the previous research results (Głaz, 2015). However, the second part of the hypothesis has not been confirmed in full. Women after stillbirth have a higher level of the presence of meaning in life than the search for meaning in life, and life satisfaction. In line with the hypothesis and previous research (Krok, 2017), it was expected that women experiencing the traumatic effects of a loss of a child show a greater interest in searching for meaning in life, i.e. they undertake a number of activities that help them interpret and understand their reality, rather than their sense of meaning in life and current satisfaction with life.

The second hypothesis, which supposes that in the life of women after a stillbirth there is a positive and stronger relationship between God’s presence than God’s absence with life satisfaction, was confirmed in full. The analysis showed that Pearson’s *r* correlation coefficient for God’s presence and life satisfaction is 0.66, and for God’s absence and life satisfaction is 0.43. And the regression coefficient for God’s presence is *β* = 0.41, and for God’s absence *β* = 0.39. This indicates that God’s presence has a stronger relationship with life satisfaction than God’s absence, and is a stronger and positive predictor of life satisfaction in the lives of women after stillbirth. This kind of relationship has already been confirmed among other respondents (Głaz, 2015).

The third hypothesis, which indicates that the experience of God’s presence and God’s absence has a stronger and more positive relationship with the search for meaning in life than with the sense of meaning in life in the lives of women after a stillbirth, has been fully confirmed. The analysis showed that the presence of God (*β* = 0.29) and the absence of God *β* = 0.31) explain 28% of the variance of the presence of meaning in life. Pearson’s correlation coefficient for the presence of God and the presence of meaning in life is 0.48, and for the absence of God and the presence of meaning in life, it is 0.31. On the other hand, the analysis revealed that the presence of God (*β* = 0.38) and the absence of God *β* = 0.37) explain 39% of the variance of the search for meaning in life. Pearson’s correlation coefficient for the search for meaning in life and the presence of God is 0.55, and for the absence of God and the search for meaning in life, it is 0.33. This confirms, in line with previous studies (Gravensteen et al., 2018; Krok, 2017), that after a traumatic experience, believing women who found themselves in a new life situation are strongly interested in searching for the meaning in their lives, interpreting and understanding the unpleasant event, while religious experience plays an important supporting role.

The fourth hypothesis suggests that the presence of meaning in life and the search for meaning in life play the role of a mediator between the presence of God and the absence of God, and life satisfaction in women after the loss of a child, has been confirmed. All analyzed variables have a significant relationship with each other. Experiencing God’s presence and God’s absence has a significant and direct impact on life satisfaction. Both variables also have an indirect impact on life satisfaction, where the presence of meaning in life and the search for meaning in life are mediating variables. The presence of God has the strongest influence on the presence of meaning in life, while with the increase in the presence of meaning in life, a greater increase in life satisfaction is visible.

Moreover, it should be added that the correlation analysis showed that in the lives of women after a stillbirth, there is a strong and positive relationship between the search for meaning in life and the presence of meaning in life (*r* = 0.57). It presumes that after losing a child, women see the meaning of their lives and at the same time taking actions that would give them a greater sense of meaning in life. Similarly, there is a stronger relationship between the presence of meaning in life and life satisfaction (*r* = 0.72) than between the search for meaning in life and life satisfaction (*r* = 0.54). This suggests that the presence of meaning in life is related to better functioning of women, satisfaction with one’s own life and experiencing its value (Popielski, 2008).

In response to painful life events, feelings such as anger toward God, feelings of rejection by God, feelings of guilt, and disruption of the religious relationship are common in the lives of religious people (Koenig, 2012; Krok, 2017). At the same time, religious faith and spirituality can play an important, positive role in the life of a person with a traumatic experience, provided they are mature (Exline & Rose, 2005). This is supported by the current research. Women of faith and practice, despite painful experiences, after the loss of a child, show trust in God, are open to the needs of others, see their lives as meaningful and valuable, and are accompanied by joy and peace. They perceive God as one who causes creative anxiety.

The loss of a child is associated with bereavement and involves spheres of psychological and religious. It manifests as grief, self-blame, low self-esteem, and sometimes loss of meaning in life (Guzewicz, 2014; Hsu et al., 2002), and often as a religious and spiritual crisis (Stelcer, 2015). Researchers point to many ways of coping with bereavement (Kersting et al., 2007; Krok, 2017). An important aspect of the bereaving process is to ensure that people are adequately assisted and helped to understand the painful event, as well as to ensure that people have the opportunity to say goodbye to their child and make a dignified burial. It can be suggested that after the stillbirth experience, Polish women, believing and practicing, have trust in God and demonstrate a constructive understanding of unpleasant and unwanted experiences, pursue life goals, perceive their lives as meaningful and valuable, and have coped well with the traumatic experience of child loss and bereavement after stillbirth.

When recalling their experiences before and after the loss of their child, many women said that with the appropriate skills and knowledge of the health service, the help they received and experienced was sufficient (Kalu, 2019; Kalu et al., 2018; Olson, 2013). Women particularly appreciated the closeness of the chaplain and the attentive listening to their problems. Some women felt that their mental health needs were neglected by health services (Guzewicz, 2014) and their immediate environment (Bellhouse, 2018; Sikora, 2014). Also, their spiritual needs were not always adequately met during their hospital stay. They expected to retain hope and have a deeper understanding of the meaning of loss (Nuzum, 2017b).

Studies show that after the loss of a child, women expect medical personnel to be more sensitive and responsive to provide specialized care, as well as to be more knowledgeable about the psychological consequences of child loss. (Bubiak et al., 2014; Gold et al., 2017). They expect the psychologist to inspire hope, encourage them to take on new tasks and appropriate strategies in difficult situations, help them accept the emotions they are experiencing, and reinforce the need for good communication with those around them (Chazan, 2013; Nuzum et al., 2017a; Olson, 2013).

An individual’s personal resources play an important role in the process of developing new forms of adaptation by individuals with traumatic experiences (Frankl, 2000; Krok et al., 2019). The literature indicates that individuals with rich personal resources find it easier to work through the experience after losing a child than those with poor resources (Stelcer, 2015). The present study shows that women who have lost a child, despite painful experiences, exhibit high levels of personal resources, i.e., search for meaning in life, presence of meaning in life, and satisfaction with life. This suggests that women respond positively to new challenges and develop new life skills and abilities (Popielski, 2008).

## Study Limitations

The current analysis of the issue raised has its limitations. Only one tool was used to study religious experience, meaning in life, and life satisfaction, respectively. Women from the northern region of Poland were examined. They were women of the Catholic faith. The studied group of women was not very large and the research was based on the convenience sample, affecting generalization to other women in Poland who were not included in the study as might be possible if this were a random sample of women experiencing stillbirth. In addition, other variables, such as age, number of children, etc., were not included in the regression analyses.

## Conclusion

The article demonstrates the problem of stillbirth and the extensive effects associated with this experience at the cognitive, emotional, and behavioral levels. It refers to the physical, psychological, and spiritual spheres. They are experienced not only by women who have suffered the loss of a child, but also by the immediate environment, which includes family, medical staff, psychologists, midwives, priests, and chaplains. In this context, the need for help and support for all is understandable.

In future studies, other tools should be used to analyze the aspects of religiousness and personality of women who have not experienced a stillbirth as a control group and compare the results with the group of women who have experienced a stillbirth. Research should also be carried out among non-believing and non-practicing women. It would be worth examining the husbands of women after the loss of a child as well as controlling some other demographic variables. Despite some limitations, the present analysis provides us with a broader view of the traumatic experience of women after the loss of a child. As researchers have noted (Guzewicz, 2014; McIntosh et al., 1993) the extent to which religious people believe that their lives are supported by a divine reality or that negative life events happen for some no fault, and if they are able to apply a religious way of dealing with these unpleasant events, they become less threatening and less stressful for them, they can be an opportunity for spiritual growth and progression of mental health.

The issue of stillbirths requires further and more extensive research to better understand the process. This requires more involvement of researchers from scientific fields, such as medicine, psychology, and religion.

With the increase in the level of education, the level of expectations of women after the loss of a child increases in relation to those associated with medical, obstetric, spiritual, and pastoral care, as well as psychological. Therefore, in the process of preparing personnel, more attention should be paid not only to the amount of expertise and skills acquired but also to the development of desirable qualities such as understanding, caring, empathetic approach, understanding, and communication with the environment.

The loss of a child has a strong impact on the psychological and spiritual life of a woman, as well as on her immediate environment. In such a situation, multifaceted help before and after the loss of a child and support for those who are involved in this painful event is of great importance. It is advisable to have more adequate cooperation between those dealing with women who have lost a child, and even to develop an appropriate method, more effective, taking into account the religious factor.

## References

[CR1] Barr P (2012). Negative self-conscious emotion and grief: An actor–partner analysis in couples bereaved by stillbirth or neonatal death. Psychology and Psychotherapy: Theory, Research and Practice.

[CR2] Bellhouse C, Temple-Smith MJ, Bilardi JE (2018). It’s just one of those things people don’t seem to talk about Women’s experiences of social support following miscarriage: a qualitative study. BMC. Women’s Health.

[CR3] Bielan Z, Machaj A, Stankowska I (2010). Psychoseksualne konsekwencje straty dziecka w okresie ciąży i porodu (Psychosexual consequences of losing a child during pregnancy and childbirth). Seksuologia Polska (polish Sexology).

[CR4] Bödecs T, Horváth B, Szilágyi E, Gonda X, Rihmer Z, Sándor J (2011). Effects of depression, anxiety, self-esteem, and health behaviour on neonatal outcomes in a population-based Hungarian sample. European Journal of Obstetrics & Gynecology and Reproductive Biology.

[CR5] Bubiak A, Bartnicki J, Knihinicka-Mercik Z (2014). Psychologiczne aspekty utraty dziecka w okresie prenatalnym (Psychological aspects of the loss of a child in the prenatal). Pielęgniarstwo i Zdrowie Publiczne (nursing and Public Health).

[CR6] Buksik, D. (2003) *Wrażliwość sumienia* (*The sensitivity of conscience*). Centrum Medyczne Pomocy Psychologiczno-Pedagogicznej.

[CR7] Burden C, Bradley S, Storey C, Ellis A, Heazell AEP, Downe S, Cacciatore J, Siassakos D (2016). From grief, guilt pain and stigma to hope and pride – a systematic review and meta-analysis of mixed-method research of the psychosocial impact of stillbirth. BMC Pregnancy and Childbirth.

[CR8] Cacciatore J, Frøen J, Killian M (2013). Condemning self, condemning other: blame and mental health in women suffering stillbirth. Journal of Mental Health Counseling..

[CR9] Cacciatore J, Ong R (2012). Through the Touch of God: Child Death and Spiritual Sustenance in a Hutterian Colony. Omega (westport).

[CR10] Campbell-Jackson L, Horsch A (2017). The Psychological Impact of Stillbirth on Women: A Systematic Review. Illness, Crisis & Loss.

[CR11] Charzyńska E (2017). Aspekty wybaczania a jakość życia uwarunkowana stanem zdrowia wśród pacjentów z nowotworem (Relationships between aspects of forgiveness and health-related quality of life in cancer patients). Humanum.

[CR12] Chazan, B. (2013). Nieudane rodzicielstwo – współczucie dla rodziców, szacunek dla ciała dziecka (Unsuccessful parenting – compassion for parents, respect for the child’s body). In J. Dziedzic (Ed.), *Od bólu po stracie do nadziei życia. Pogrzeb dziecka poronionego* (*From pain after loss to hope for life. Funeral of a miscarried child*). Uniwersytet Papieski JP II w Krakowie. pp. 207–214

[CR13] Cowchock FS, Lasker JN, Toedter LJ, Skumanich SA, Koenig HG (2010). Religious beliefs affect grieving after pregnancy loss. Journal of Religion and Health.

[CR14] Cuisinier MCJ, Kuijpers JC, Hoogduin CAL, de Graauw CPH, Janssen HJE (1993). Miscarriage and stillbirth: Time since the loss, grief intensity and satisfaction with care. European Journal of Obstetrics & Gynecology and Reproductive Biology.

[CR15] DeFrain J (1991). Learning about grief from normal families: SIDS, stillbirth, and miscarriage. Journal of Marital and Family Therapy.

[CR16] Diener E, Chan MY (2011). Happy People Live Longer: Subjective Well-Being Contributes to Health and Longevity. Applied Psychology: Health and Well-Being.

[CR17] Diener E, Emmons RA, Larsen RJ, Griffin S (1985). The Satisfaction with Life Scale. Journal of Personality Assessment.

[CR18] Downey G, Silver RC, Wortman CB (1990). Reconsidering the attribution-adjustment relation following a major negative event: Coping with the loss of a child. Journal of Personality and Social Psychology.

[CR19] Dyrdal G, Røysamb E, Nes R, Vittersø J (2011). Can a happy relationship predict a happy life? A Population-based study of maternal well-being during the life transition of pregnancy, infancy, and toddlerhood. Journal of Happiness Studies.

[CR20] Emond T, de Montigny F, Guillaumie L (2019). Exploring the needs of parents who experience miscarriage in the emergency department: A qualitative study with parents and nurses. Journal of Clinical Nursing.

[CR21] Exline JJ, Rose E, Paloutzian RF, Park CL (2005). Religious and Spiritual Struggles. Handbook of the Psychology of Religion and Spirituality.

[CR22] Frankl VE (2000). Man’s search for ultimate meaning.

[CR23] Giannandrea SA, Cerulli C, Anson E, Chaudron LH (2013). Increased risk for postpartum psychiatric disorders among women with past pregnancy loss. Journal of Women’s Health.

[CR24] Głaz S (2015). The Importance of the Experience of God’s Absence, and of Meaning in Life, in the development of Sensitivity of Conscience among Polish University Students. Religions.

[CR25] Głaz S (2021). Psychological Analysis of Religious Experience: The Construction of the Intensity of Religious Experience Scale (IRES). Journal of Religion and Health.

[CR26] Gold KJ, Sen A, Leon I (2017). Whose Fault Is It Anyway? Guilt, Blame, and Death Attribution by Mothers After Stillbirth or Infant Death. Illness, Crisis & Loss.

[CR27] Gravensteen IK (2017) Stillbirth: Women´s long-term quality of life, mental health and the subsequent pregnancy. Results from two observational studies on women with a history of stillbirth. Department of Haematology, Oslo University Hospital Institute of Basic Medical Sciences and Institute of Clinical Medicine, University of Oslo. February, 2017.

[CR28] Gravensteen IK, Jacobsen EM, Sandset PM, Helgadottir LB, Rådestad I, Sandvik L, Ekeberg Ø (2018). Anxiety, depression and relationship satisfaction in the pregnancy following stillbirth and after the birth of a live-born baby: A prospective study. BMC Pregnancy and Childbirth.

[CR29] Guzewicz M (2014). Psychologiczne i społeczne konsekwencje utraty dziecka w wyniku poronienia (Psychological and social consequences of losing a child through stillbirth). Civitas Et Lex.

[CR30] Guzewicz M, Steuden S, Szymona-Pałkowska K (2014). Changes in the perception of self-image and the sense of purpose and meaning in life, among women who lost their child before birth. Health Psychology Report..

[CR31] Guzikowski W, Lichtenberg-Kokoszka E, Janiuk E, Dzierżanowski J (2009). Wybrane zagadnienia i aspekty niepłodności kobiecej (Selected issues and aspects of female infertility). Niepłodność – zagadnienie interdyscyplinarne (Infertility – an interdisciplinary issue).

[CR32] Hamama-Raz Y, Hartman H, Buchbinder E (2014). Coping With Stillbirth Among Ultraorthodox Jewish Women. Qualitative Health Research.

[CR33] Hsu MT, Tseng YF, Kuo LL (2002). Transforming loss: Taiwanese women’s adaptation to stillbirth. Journal of Advanced Nursing.

[CR34] John Paul II (1983). *Code of Canon Law*. Vatican Publishing House.

[CR35] Juczyński, Z. (2001). *Narzędzia pomiaru w promocji i psychologii zdrowia* (*Measurement tools in promotion and health psychology*). Pracownia Testów Psychologicznych Polskiego Towarzystwa Psychologicznego.

[CR36] Kalu FA (2019). Women’s experiences of utilizing religious and spiritual beliefs as coping resources after miscarriage. Religions.

[CR37] Kalu FA, Coughlan B, Larkin Ph (2018). A mixed methods sequential explanatory study of the psychosocial factors that impact on midwives’ confidence to provide bereavement support to parents who have experienced a perinatal loss. Midwifery.

[CR38] Kersting A, Kroker K, Steinhard J, Lüdorff K, Wesselmann U, Ohrmann P, Arolt V, Suslow T (2007). Complicated grief after traumaticloss: A 14-month follow up study. European Archives of Psychiatry and Clinical Neuroscience.

[CR39] Kersting A, Wagner B (2012). Complicated grief after perinatal loss. Dialogues in Clinical. Neuroscience.

[CR40] Koenig HG (2012) Religion and mental health. In: HG Koenig, DE King, B Carson Verna (Eds) *Handbook of religion and health*. Oxford University Press: Oxford. pp 109–624

[CR41] Kossakowska M, Kwiatek P, Stefaniak T (2013). Sens w życiu Polska wersja kwestionariusza MLQ (Meaning in Life Questionnaire). Psychologia Jakości Życia (quality of Life Psychology)..

[CR42] Krok D. (2017). *W poszukiwaniu znaczenia choroby nowotworowej* (*In search of the meaning of cancer*). Uniwersytet Opolski.

[CR43] Krok D (2015). The role of meaning in life within the relations of religious coping and psychological well-being. Journal of Religion and Health.

[CR44] Krok D, Brudek P, Steuden S (2019). When meaning matters: Coping mediates the relationship of religiosity and illness appraisal with well-being in older cancer patients. The International Journal for the Psychology of Religion.

[CR45] Lamb EH (2002). The impact of previous perinatal loss on subsequent pregnancy and parenting. Journal of Perinatal Education.

[CR46] Lawn JE, Gravett MG, Nunes TM, Rubens CE, Stanton C (2010). GAPPS Review Group Global report on preterm birth and stillbirth (1of7): definitions, description of the burden and opportunities to improve data. BMC Pregnancy and Childbirth.

[CR47] Mann JR, Mannan J, Quiñones LA, Palmer AA, Torres M (2010). Religion, spirituality, social support, and perceived stress in pregnant and postpartum Hispanic women. Journal of Obstetrics & Gynecology and Neonatal Nursing.

[CR48] Mann JR, McKeown RE, Bacon J, Vesselinov R, Bush F (2008). Do antenatal religious and spiritual factors impact the risk of postpartum depressive symptoms?. Journal of Women’s Health.

[CR49] McIntosh DN, Silver RC, Wortman CB (1993). Religion’s role in adjustment to a negative life event: Coping with the loss of a child. Journal of Personality and Social Psychology.

[CR50] Merrill R, Read C, LeCheminant A (2009). The influence of religiosity on positive and negative outcomes associated with stress among college students. Mental Health, Religion & Culture.

[CR51] Miernik B (2017). Poronienie samoistne jako doświadczenie rodzinne – psychopedagogiczne aspekty straty dziecka w okresie prenatalnym (Spontaneous stillbirth as a family experience – psychopedagogical aspects of the loss of a child in the prenatal period). Fides Et Ratio.

[CR52] Nematti A (2005). Abortion in the views of contemporary Islamic jurisprudence. Journal of Reproduction and Infertility.

[CR53] Newitt M (2014). Chaplaincy support to bereaved parents – part 1. Health and Social Care Chaplaincy.

[CR54] Nuzum D, Meaney S, O’Donoghue K (2016). The Place of faith for consultant obstetricians following stillbirth: A qualitative exploratory study. Journal of Religion and Health.

[CR55] Nuzum D, Meaney S, O’Donoghue K (2017). The Spiritual and Theological Challenges of Stillbirth for Bereaved Parents. Journal of Religion and Health.

[CR56] Nuzum D, Meaney S, O’Donoghue K (2018). The impact of stillbirth on bereaved parents: A qualitative study. PLoS ONE.

[CR57] Nuzum D, Meaney S, O’Donoghue K, Jackson M (2017). Stillbirth and suffering in Ireland: A theological reflection from healthcare chaplaincy. Practical Theology.

[CR58] Okła W, Steuden S, Janowski K (2009). Człowiek w sytuacji choroby (Man in a sick situation). Psychospołeczne konteksty doświadczania straty (Psychosocial contexts of experiencing loss).

[CR59] Olson KJ (2013). Health promotion. Healing through loss. Journal of Emergency Nursing.

[CR60] Pantke R, Slade P (2006). Remembered parenting style and psychological well-being in young adults whose parents had experienced early child loss. Psychology and Psychotherapy.

[CR61] Pargament KI (1997). The psychology of religion and coping: Theory, research, practice.

[CR62] Pilecka, B. (2004). *Kryzys psychologiczny. Wybrane zagadnienia* (*Psychological crisis. Selected Issues*). Wydawnictwo UJ.

[CR63] Podolska, M. (2011). *Niepłodność i jej następstwa psychologiczne. Stan badań i perspektywy* (*Infertility and its psychological consequences. State of research and prospects*). Wydawnictwo Naukowe Uniwersytetu Szczecińskiego: Lublin

[CR64] Popielski, K (2008) *Psychologia egzystencji. Wartości w życiu* (*Psychology of Existence. Values in life*) KUL. Wydawnictwo Katolickiego Uniwersytetu Lubelskiego: Lublin. pp 17–64

[CR65] Prusak J, Kwapis K, Pilecka B, Chemperek A, Krawczyk A, Jabłoński M, Nowakowski K (2021). The quality of life, meaning in life, positive orientation to life and gratitude of catholic seminarians in Poland: A comparative analysis. Archive for the Psychology of Religion.

[CR66] Rådestad I, Sjögren B, Nordin C, Steineck G (1997). Stillbirth and maternal well-being. Acta Obstetricia Et Gynecologica Scandinavica.

[CR67] Roberts LR, Montgomery SB (2015). Mindfulness-based Intervention for Perinatal Grief after Stillbirth in Rural India. Issues in Mental Health Nursing.

[CR68] Roesch SC, Ano G (2003). Testing an Attribution and Coping Model of Stress: Religion as an Orienting System. Journal of Psychology and Christianity.

[CR69] Sikora K (2014). Reakcje kobiet po stracie ciąży oraz zachowania partnerów (The reaction of women after losing their child while pregnant with it and their partners’ behaviours). Polish Scientific Journal Database.

[CR70] Steger MF, Frazier P, Oishi S, Kaler M (2006). The Meaning in Life Questionnaire: Assessing the Presence of and Search for Meaning in Life. Journal of Counseling Psychology.

[CR71] Stelcer B (2015). Żal po stracie – dynamika adaptacji do nieuniknionych zmian (Grief after loss – process of adjustment to loss and change). Sztuka Leczenia (the Art of Treatment).

[CR72] Thearle MJ, Vance JC, Najman JM, Embelton G, Foster WJ (1995). Church attendance, religious affiliation and parental responses to sudden infant death, neonatal death and stillbirth. OMEGA - Journal of Death and Dying.

[CR73] Dziennik Ustaw, (2020) poz. 666. Rozporządzenie Ministra Zdrowia z dnia 6 kwietnia 2020 r w sprawie rodzajów, zakresu i wzorów dokumentacji medycznej oraz sposobu jej przetwarzania. (Legislative Bulletin, 2020 pt. 666. Regulation of the Minister of Health of 6 April 2020 on types, scope and models of medical records and the manner of their processing).

[CR74] Van den Akker O (2011). The psychological and social consequences of miscarriage. Expert Review of Obstetrics and Gynecology.

[CR75] Vance JC, Foster WJ, Najman JM, Embelton G, Thearle MJ, Hodgen FM (1991). Early parental responses to sudden infant death, stillbirth, or neonatal death. The Medical Journal of Australia.

[CR76] Weinberg N (1995). Does apologizing help? The role of self-blame and making amends in recovery from bereavement. Health & Social Work.

[CR77] Wonch Hill P, Cacciatore J, Shreffler KM, Pritchard KM (2017). The loss of self: The effect of miscarriage, stillbirth, and child death on maternal self-esteem. Death Studies.

[CR78] World Health Organization. World Health Organization; Geneva 2004. (International statistical classification of diseases and related health problems ICD-10, 10th revision) vol. 2. http://apps.who.int/gho/indicatorregistry/App_Main/view_indicator.aspx?iid=2444

